# Modulatory effect of protocatechuic acid on cadmium induced nephrotoxicity and hepatoxicity in rats in vivo

**DOI:** 10.1186/s40064-015-1408-6

**Published:** 2015-10-16

**Authors:** Stephen A. Adefegha, Olasunkanmi S. Omojokun, Ganiyu Oboh

**Affiliations:** Functional Foods and Nutraceuticals Unit, Department of Biochemistry, Federal University of Technology, P.M.B. 704, Akure, 340001 Ondo State Nigeria

**Keywords:** Cadmium, Hepatotoxicity, Nephrotoxicity, Protocatechuic acid

## Abstract

**Introduction:**

This study sought to investigate the effect of protocatechuic acid (PCA); a phenolic compound readily available in most plant foods on cadmium-induced nephrotoxicity and hepatoxicity in rats.

**Case description:**

Thirty six adult male rats weighing about 150–160 g were acclimatized for 2 weeks and subsequently divided into six groups: Group 1 rats received normal saline (control group), group 2 rats were administered 5 mg Cd/kg body weight in form of solution orally (induced group), groups 3 and 4 received cadmium solution and different doses of PCA (10 and 20 mg/kg body weight) respectively, while groups 5 and 6 were the normal rats administered different doses of PCA (10 and 20 mg/kg) respectively in an experiment that lasted for twenty one days. The animals were sacrificed, the blood was collected and the serum was subsequently prepared. Furthermore, the liver was excised, homogenized and centrifuged to obtain the tissue homogenate used for the analyses. The serum was used for the determination of the total protein, urea, creatinine and uric acid levels while the liver homogenate was used for the estimation of alanine aminotransferase (ALT), aspartate transaminase (AST), and alkaline phosphatase (ALP).

**Discussion and evaluation:**

The result revealed that total protein level was reduced in cadmium induced toxicity rat group which was elevated upon treatment with PCA. Conversely, the elevated levels of urea, uric acid and creatinine in cadmium induced toxicity kidney rats were significantly (p < 0.05) reduced in PCA treated groups. Similarly, marked elevation in the ALT, AST and ALP activity were observed in cadmium induced toxicity rat group when compared with the control group. However, significant (p < 0.05) decrease in ALT, AST and ALP activity were noticed in groups administered different doses of PCA.

**Conclusions:**

The results from this study suggest that PCA may protect against cadmium-induced toxicity in the kidney and liver.

## Background

Cadmium (Cd) is a heavy metal obtained as a by-product of zinc. It is one of the most toxic environmental and industrial pollutants, obtained through consumption of foods and drinking water, inhaled from air or cigarette smoking or from ingestion of contaminated soil and dust. It is a long biological half-life, toxic and non-biodegradable metal that is non-beneficial to plants, animals and humans (Jarup et al. 2000). The most important target organ for chronic low-level exposure to Cd is the kidney and is reflected in proteinuria, calciuria, aminoaciduria, glycosuria and tubular necrosis (Satarug et al. 2010) which may eventually lead to end-stage renal failure, deregulated blood pressure, diabetic complications and osteoporosis (Satarug and Moore 2004). In addition, Cd has the propensity to settle in the proximal tubule of the nephron, leading to nephrotoxicity (Ahn et al. 1999).

Cd shows different mechanisms of toxicity under different experimental conditions and in various species. When some toxicants enter into the human system they result in disturbance in the redox balance in cells in favour of oxidants, with the imbalance resulting in oxidative stress which eventually results in oxidative damage to the cellular macromolecules and cellular structures (Waisberg et al. 2003). Although, the biochemical mechanism behind Cd nephrotoxicity and hepatotoxicity is not perfectly understood, once taken up enterally, Cd reaches the liver where it binds to metallothioneins (MTs), glutathione (GSH) and other proteins or peptides (Thevenod 2009). Even though, mitochondria have been identified as the earliest target organelles of Cd-induced nephrotoxicity, antioxidants are known to protect against oxidative stress and Cd-induced nephrotoxicity (Tang and Shaikh 2001).

Protocatechuic acid (PCA) which is chemically known as 3, 4-hydroxybenzoic acid is a widely distributed naturally occurring phenolic acid and also one of the main metabolites of complex polyphenols which are normally found at high concentrations in vegetables and fruit, and are readily absorbed by animals and humans (Vitaglione et al. 2007). Protocatechuic acid has multifaceted biological effects by acting on different molecular targets as it is shown to possesses antioxidant, anti-inflammatory as well as anti-hyperglycemic activities (Lin et al. 2009). However, there are new insights in the comprehension of the cellular and molecular mechanisms responsible for the potential preventive/therapeutic activity of PCA against a number of human diseases, but there is a dearth of information on its nephro-protective and hepato-protective mechanism. Hence we propose in this study to investigate possible modulatory effect of Protocatechuic acid on liver marker enzymes and kidney parameters in cadmium-induced hepatotoxicity and nephrotoxicity in rats in vivo.

## Materials and case descriptions

### Materials

Kits used for bioassay (total protein, urea, uric acid, creatinine, ALT, AST and ALP) were sourced from Randox Laboratories Ltd., Crumlin, County Antrim, UK. Except where otherwise stated, all chemicals used were sourced from Sigma Co. (St Louis MO, USA). The water used was glass distilled.

#### Experimental animal and treatment groups

Thirty six adult male Wistar rats weighing about 150–160 g were purchased from Central Animal House University of Ibadan, Oyo state. In this work, six groups were taken; each group comprised six male healthy rats. The first group served as control group with a normal feed and normal saline which is used as the positive control group, the induced group (group II) received cadmium chloride only orally (5 mg of Cd/kg of body weight in form of solution) which is the negative control. The third group received cadmium and 10 mg/kg PCA, the fourth group received cadmium and 20 mg/kg PCA, the fifth and sixth groups received (10 and 20 mg/kg body weight) PCA respectively. The rats were selected and kept in controlled conditions of about 12 h dark and 12 h light (alternative dark light cycle 12:12). All the animals were fed with a standard laboratory feed and water ad libitum. Care and treatment of the animals were performed accordingly. All the procedure were carried out orally. At the end of the experimental period, the animals were anaesthetized and sacrificed after overnight fast.

### Animal ethics

The male Wistar rats used received humane care according to the criteria outlined in the Guide for the Care and the Use of Laboratory Animals prepared by EU Directive 2010/63/EU for animal experiments. The ethic regulations have been followed in accordance with national and institutional guidelines for the protection of animals’ welfare during experiments. The experiment was carried out at the Functional Food, Nutraceuticals and Phytomedicine Laboratory, Department of Biochemistry, Federal University of Technology, Akure, Ondo State, Nigeria.

#### Homogenate preparation and Serum sample analysis

The rats were decapitated via cervical dislocation and the liver was rapidly dissected, placed in phosphate buffer pH 7.4 on ice and weighed. This tissue was subsequently rinsed with the phosphate buffer pH 7.4 and later homogenized with the phosphate buffer pH 7.4 (1:5 w/v), with about 10-up and down strokes at approximately 1200 rev/min in a Teflon-glass homogenizer. The homogenate was centrifuged for 10 min at 3000 g to yield a pellet that was discarded and the supernatant was used for ALT, AST and ALP assay.

Function of kidney was assessed by measuring the concentration of total protein, urea, uric acid and creatinine in serum.

### Methods

#### Determination of serum total protein concentration

The total protein concentration was determined using the method described by Lowry et al. 10 µl of distilled water was pipetted into the test tube labeled blank, 10 µl of standard was added to the test tube labeled standard, and 10 µl of the sample was added to the sample test tube. 500 µl of Reagent was added to the three test tubes. The solution was mixed and incubated for 30 min at 25 °C after which absorbance was read at 540 nm (Lowry et al. 1951).

#### Determination of serum uric acid concentration

The uric acid concentration was determined using spectrophotometric method described by Collin and Diehl with slight modification by Morin and Prox. Briefly, 20 μL of distilled water was added to 20 μL of the sample which was mixed with 1 mL of Hepes reagent (50 mM phosphate buffer, 4 mM 3,5-chloro-2-hydroxybenzenesulfonic acid) and enzyme reagent (0.25 mM 4-aminophenazone, peroxidise, and uricase). Thereafter, the mixture was incubated for 5 min at 37 °C and the absorbance at 520 nm was taken against reagent blank within 30 min (Collin and Diehl 1959; Morin and Prox 1973). The uric acid concentration was subsequently calculated against the standard.

#### Determination of serum urea concentration

The urea concentration was determined using spectrophotometric method described by Searcy et al. Briefly, 10 μL of sample was added to 0.1 mL of sodium nitroprusside—urease reagent (116 mM EDTA, 6 mM sodium nitroprusside, 1 g/L urease) after which the mixture was incubated for 10 min at 37 °C. 2.5 mL of 120 mM diluted phenol and 2.5 mL of 27 mM sodium hypochlorite solution containing 0.14 N sodium hydroxide which was then added to the reaction mixture. Thereafter, the mixture was incubated for 15 min at 37 °C and the absorbance at 546 nm was taken against reagent blank within 8 h (Searcy et al. 1967). The urea concentration was subsequently calculated against the standard.

#### Determination of serum creatinine concentration

The creatinine concentration was determined using spectrophotometric alkaline Jaffe picrate method as described by Spierto et al. Briefly, 50 μL of distilled water was added to 2 mL of working reagent (35 mM picric acid and 0.32 M sodium hydroxide) before 50 μL of sample was added. Thereafter, the mixture was allowed to stay for 30 s before taking absorbance. The absorbance at 492 nm was taken twice, firstly after 30 s and secondly after 2 min (Spierto et al. 1979). The creatinine concentration was subsequently calculated against the standard, using change in the sample absorbance.

#### Determination of aspartate transaminase (AST) activity

This was carried out according to the method of Reitman and Frankel as described by the manufacturer’s manual (Randox Laboratories Ltd). Briefly, 100 μl of test sample was mixed with 500 μl of buffer (containing 100 mM phosphate buffer pH 7.4, 100 mM l-aspartate, and 2 mM α-oxoglutarate) and the mixture was incubated for 30 min at 37 °C. Thereafter, 500 μl of 2 mM 2,4 dinitrophenylhydrazine was added to the reaction mixture and allowed to stand for 20 min at 25 °C. Then, 500 μl of 0.4 mM NaOH was added and thoroughly mixed; the absorbance was read after 5 min at 546 nm against a reagent blank and the AST activity determined (Reitman and Frankel 1957).

#### Determination of alanine aminotransferase (ALT) activity

This was carried out according to the method of Reitman and Frankel as described by the manufacturer’s manual (Randox Laboratories Ltd). Briefly, 100 μl of test sample was mixed with 500 μl of buffer (containing 100 mM phosphate buffer pH 7.4, 200 mM l-alanine and 2 mM α-oxoglutarate) and the mixture was incubated for 30 min at 37 °C. Thereafter, 500 μl of 2 mM 2, 4 dinitrophenylhydrazine was added to the reaction mixture and allowed to stand for 20 min at 25 °C. Then, 500 μl of 0.4 mM NaOH was added and thoroughly mixed; the absorbance was read after 5 min at 546 nm against a reagent blank and the AST activity determined (Reitman and Frankel 1957).

#### Determination of alkaline phosphatase activity (ALP)

The serum ALP concentration was determined using spectrophotometric method according to the recommendations of Deutsche Gesellschaft fur Klinische Chemie,. Briefly, 20 μl of the test sample was mixed with 1 ml of reacting mixture (containing 1 M Diethanolamine buffer pH 9.8, 0.5 mM MgCl_2_ and 10 mM *p*-nitrophenylphosphate). The absorbance was then read at 1 min interval for 3 min at 405 nm and the ALP activity was subsequently determined (DGKC 1972).

### Data analysis

The results of replicate readings were pooled and expressed as mean ± standard deviation. Unpaired Student’s *t* test was used for analyzing the data between two groups, whereas one-way analysis of variance (ANOVA) followed by multiple comparison tests (Tukey’s test) was employed for the post hoc (Zar 1984). GraphPad prism 6 software package for Windows was used for the analysis. The level of significance was accepted at *P* < 0.05 for the assessment of in vivo biochemical parameters.

## Case presentation

**Fig. 1:**
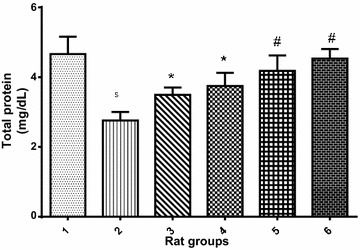
Effect of protocatechuic acid on total protein concentration in the serum of rats exposed to cadmium. ^$^Group 2 (Cadmium-induced toxicity) significantly (p < 0.05) different from group 1 (normal control). *Group 3 (Cadmium + 10 mg/kg B.W PCA) and 4 (Cadmium + 20 mg/kg B.W PCA) significantly (p < 0.05) different from group 2 (Cadmium-induced toxicity). ^#^Group 5 (10 mg/kg B.W PCA) and 6 (20 mg/kg B.W PCA) not significantly (p > 0.05) different from group 1(normal control)

The effect of protocatechuic acid (PCA) on the protein level in rat serum induced with cadmium (5 mg of Cd/kg B.W) as shown in Fig. 1 revealed that there was significant (p < 0.05) reduction in protein level of cadmium-treated rat group when compared with the normal control group. However, co-treatment of cadmium with 10 or 20 mg/kg PCA significantly (p < 0.05) elevated the protein level. Also, administration of PCA (10 or 20 mg/kg) resulted in the elevation of the protein level with no significant (p > 0.05) difference when compared with normal control.

**Fig. 2:**
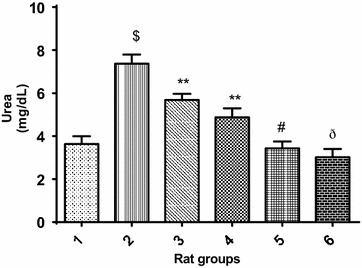
Effect of protocatechuic acid on urea level in the serum of rats exposed to cadmium. ^$^Group 2 (Cadmium-induced toxicity) significantly (p < 0.05) different from group 1 (normal control). **Group 3 (Cadmium + 10 mg/kg B.W PCA) and 4 (Cadmium + 20 mg/kg B.W PCA) very significantly (p < 0.05) different from group 2 (Cadmium-induced toxicity). ^#^Group 5 (10 mg/kg B.W PCA) not significantly (p > 0.05) different from group 1 (normal control). ^ð^Group 6 (20 mg/kg B.W PCA) significantly (p < 0.05) different from group 1 (normal control)

**Fig. 3:**
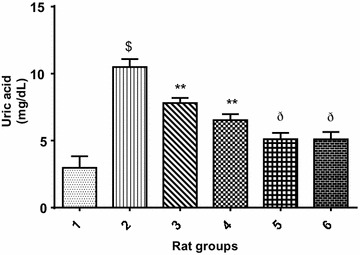
Effect of protocatechuic acid on uric acid level in the serum of rats exposed to cadmium. ^$^Group 2 (Cadmium-induced toxicity) significantly (p < 0.05) different from group 1 (normal control). **Group 3 (Cadmium + 10 mg/kg B.W PCA) and 4 (Cadmium + 20 mg/kg B.W PCA) very significantly (p < 0.05) different from group 2 (Cadmium-induced toxicity). ^ð^Group 5 (10 mg/kg B.W PCA) and 6 (20 mg/kg B.W PCA) significantly (p < 0.05) different from group 1 (normal control)

**Fig. 4:**
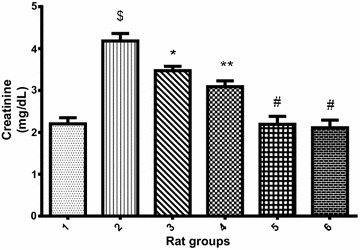
Effect of protocatechuic acid on creatinine level in the serum of rats exposed to cadmium. ^$^Group 2 (Cadmium-induced toxicity) significantly (p < 0.05) different from group 1 (normal control). *Group 3 (Cadmium + 10 mg/kg B.W PCA) significantly (p < 0.05) different from group 2 (Cadmium-induced toxicity). **Group 4 (Cadmium + 20 mg/kg B.W PCA) very significantly (p < 0.05) different from group 2 (Cadmium-induced toxicity). ^#^Group 5 (10 mg/kg B.W PCA) and 6 (20 mg/kg B.W PCA) not significantly (p > 0.05) different from group 1(normal control)

Furthermore, Fig. 2 revealed that cadmium administration significantly (p < 0.05) increased the urea level in cadmium-treated rats when compared with the normal control group. However, co-treatment of cadmium with 10 or 20 mg/kg PCA (Group 3 and 4 respectively) showed obvious significant (p > 0.05) decrease in urea level. In addition, treatment with 10 or 20 mg/kg PCA (Group 5 and 6 respectively) significantly (p < 0.05) reduced the urea level when compared with cadmium–treated group. Similarly, there was a significant decrease (p < 0.05) in urea level in normal rat treated with PCA (10 and 20 mg/kg) when compared with the cadmium-treated group (Fig. 2). Although, the decrease in urea level of the 20 mg/kg PCA group has slight significant (p < 0.05) difference from the normal control group, this could ascertain the 20 mg/kg as the fixed dose for further biochemical studies. Similarly, Fig. 3 revealed that cadmium administration significantly (p < 0.05) increased the uric acid level in cadmium-treated group when compared with the normal control group. However, co-treatment of cadmium with 10 or 20 mg/kg PCA showed significant (p > 0.05) decrease in uric acid level. In addition, there was a significant difference (p < 0.05) in uric acid level in normal rat treated with PCA (10 and 20 mg/kg) when compared with the cadmium-treated group as well as with normal control group. Also, Cd administration significantly (p < 0.05) increased the creatinine level in Cd-treated negative control group when compared with the normal control group. However, there was a significant decrease (p < 0.05) in creatinine level of rats in the groups co-treated with cadmium and PCA (10 and 20 mg/kg) when compared with the cadmium-induced toxicity group (Fig. 4). Furthermore, no significant (p > 0.05) change was observed between the PCA (10 and 20 mg/kg) treated and normal control groups.

**Fig. 5:**
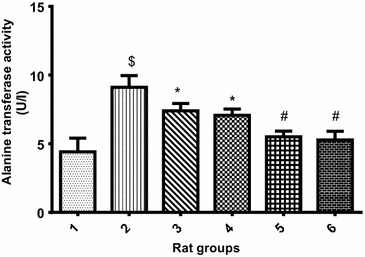
Effect of protocatechuic acid on ALT activity in the liver of rats exposed to cadmium. ^$^Group 2 (Cadmium-induced toxicity) significantly (p < 0.05) different from group 1 (normal control). *Group 3 (Cadmium + 10 mg/kg B.W PCA) and 4 (Cadmium + 20 mg/kg B.W PCA) significantly (p < 0.05) different from group 2 (Cadmium-induced toxicity). ^#^Group 5 (10 mg/kg B.W PCA) and 6 (20 mg/kg B.W PCA) not significantly (p > 0.05) different from group 1(normal control)

**Fig. 6:**
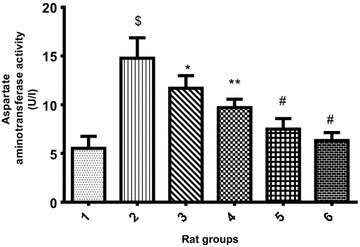
Effect of protocatechuic acid on AST activity in the liver of rats exposed to cadmium. ^$^Group 2 (Cadmium-induced toxicity) significantly (p < 0.05) different from group 1 (normal control). *Group 3 (Cadmium + 10 mg/kg B.W PCA) significantly (p < 0.05) different from group 2 (Cadmium-induced toxicity). **Group 4 (Cadmium + 20 mg/kg B.W PCA) very significantly (p < 0.05) different from group 2 (Cadmium-induced toxicity). ^#^Group 5 (10 mg/kg B.W PCA) and 6 (20 mg/kg B.W PCA) not significantly (p > 0.05) different from group 1(normal control)

**Fig. 7:**
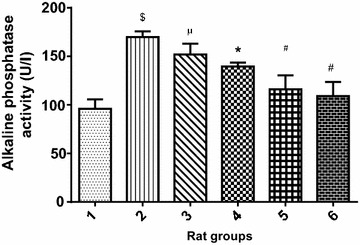
Effect of protocatechuic acid on ALP activity in the liver of rats exposed to cadmium. ^$^Group 2 (Cadmium-induced toxicity) significantly (p < 0.05) different from group 1 (normal control). ^μ^Group 3 (Cadmium + 10 mg/kg B.W PCA) not significantly (p > 0.05) different from group 2 (Cadmium-induced toxicity). *Group 4 (Cadmium + 20 mg/kg B.W PCA) significantly (p < 0.05) different from group 2 (Cadmium-induced toxicity). ^#^Group 5 (10 mg/kg B.W PCA) and 6 (20 mg/kg B.W PCA) not significantly (p > 0.05) different from group 1(normal control)

Figures 5, 6 and 7 displayed the effect of PCA on ALT, AST and ALP respectively. The liver enzyme activity in cadmium-induced hepatotoxicity in Wistar rats revealed that there was significant (p < 0.05) increase in ALT, AST and ALP activity of Cd-treated rat group when compared with the normal control group. However, the groups co-treated with cadmium and 10 or 20 mg/kg PCA significantly (p < 0.05) reduced the liver enzymes activity. Conversely, there was no significant (p > 0.05) difference in the enzymes activity following administration of PCA (10 or 20 mg/kg) when compared with the normal control group.

## Discussion

Protocatechuic acid (PCA) has attracted the interest of researchers in the last few years because of the antioxidant activity. However, the possible mechanisms of action of PCA in liver and kidney protection have not been fully investigated. In this study, the significantly (p < 0.05) reduced total protein level in the Cd-treated rat group when compared with the normal control rats may be attributed to the oxidation of protein thereby forming protein-adduct leading to the oxidative damage. However, the significant (p < 0.05) elevation in the total protein level of Cd-treated rat group co-treated with PCA and also the normal rat groups treated with PCA may be as a result of injury on renal and hepatic cells induced by Cd intoxication which subsequently disturbed protein biosynthesis (Chawla 2003). However, this effect was more profound and effective in the PCA only treated group indicating the potential of PCA to regenerate damaged renal and hepatic tissue, and thus increased protein synthesis in the damaged tissues (Salama and El-Bahr 2007).

The increased levels of serum urea, uric acid and creatinine in the Cd-treated rat group may be attributed to oxidative imbalance in the kidney causing elevated urea, uric acid and creatinine in the blood. Findings from this study correlate with the report given by Gilrolami et al. where urea level was elevated as a result of Cd induction (Girolami et al. 1989). The significantly (p < 0.05) elevated level of creatinine in Cd-treated rat group when compared with the normal rat group may be attributed to the oxidative damage to the kidney thereby allowing more release of creatinine into the blood. This is in agreement with earlier reports by Renugadevi and Prabu where Cd administration leads to an elevated level of creatinine (Renugadevi and Prabu 2009). This was also linked to the defect in filtration. Also, Shatti reported that rise in creatinine level is an indication of renal–tubular damage due to Cd-induced nephrotoxicity (Shatti 2011). The reduction of the elevated urea, uric acid and creatinine level by cadmium may suggest that PCA exerts nephro-protective effect when exposed to Cd.

ALT and AST are considered to be established indicators of hepatocellular damage (Al-Habori et al. 2002). As a result of antioxidants/oxidants imbalance ratio in the cells, the levels of hepatic enzymes (ALT, AST and ALP) activities may indicate liver tissue damage probably by altered cell membrane permeability leading to the leakage of the enzymes from the tissue to the serum (Gaskill et al. 2005). In this study, a severe hepatic damage was observed by the significant elevation of the hepatic enzymes ALT, AST and ALP activity in the serum of the Cd-induced hepatotoxicity group when compared with the normal control group. Finding from this study is similar to previously reported work by Soufy et al. who reported the hepatotoxic effect of Cd in rats exposed to the metal (Renugadevi and Prabu 2009). They further affirmed that these changes may be due to direct toxic effects of the toxicant (Cd) on hepatocytes since the liver is the site of detoxification of all types of toxins and chemicals (Renugadevi and Prabu 2009; Soufy et al. 2007). The liver being the organ mostly associated with the detoxification and biotransformation of most foreign compounds that enter the body gets its regulating mechanism impaired as a result of accumulated toxicants which could result to the tissue damage (Camargo and Martinez 2006). This is in agreement with a report by Liu et al. that PCA exerts a potent attenuating effect on fat-induced liver damage (Liu et al. 2010). Similarly, various studies have reported the in vitro antioxidant potential, antibacterial effect and cancer chemopreventive activity of PCA (Li et al. 2011; Chao and Yin 2009; Tanaka et al. 2011) showing its general pharmacological relevance. In this study, accumulation of Cd in the liver could be deployed to be what accounted for the hepatocellular injury noticed. Interestingly, serum levels of the renal functional markers and hepatic enzymes activities measured in this study were ameliorated upon co-treatment of Cd and PCA while no adverse effect was noticed following PCA administration in rats suggesting a safe use of this plant based phenolic compound and the dose used in this study on the kidney and the liver.

## Conclusion

From this study, it can be concluded that Cd induction of lipid peroxidation and elevation of liver marker enzymes and kidney parameters in the serum may interfere with the cellular balance in the kidney and liver thereby causing oxidative damage. However, PCA modulated Cd-induced nephrotoxicity and hepatotoxicity in rats.
